# From Polyethylene to Highly Graphitic and Magnetic Carbon Spheres Nanocomposites: Carbonization under Pressure

**DOI:** 10.3390/nano9040606

**Published:** 2019-04-12

**Authors:** Jesica Castelo-Quibén, Luisa M. Pastrana-Martínez, Francisco Carrasco-Marín, Agustín F. Pérez-Cadenas

**Affiliations:** Carbon Materials Research Group, Department of Inorganic Chemistry, Faculty of Sciences, University of Granada, Campus Fuentenueva s/n, ES18071-Granada, Spain; lpastrana@ugr.es (L.M.P.-M.); fmarin@ugr.es (F.C.-M.); afperez@ugr.es (A.F.P.-C.)

**Keywords:** carbon microspheres, metal-carbon nanocomposites, magnetic composites

## Abstract

Carbon nanocomposites microspheres were synthesized from Low-Density Polyethylene (LDPE) by a facile one-step strategy under solvent-free conditions. The synthesis of these materials was carried out in a closed Hastelloy^®^ reactor at 700 °C. The treatment, during which autogenic pressure was generated, leads to highly graphitic materials with stunning properties, particularly concerning the oxidation resistance (compared to the graphite stability). The metallic doping triggers the growth of nanostructures with diverse morphologies around the spheres, obtaining samples with magnetic properties.

## 1. Introduction

Carbon materials are excellent candidates to be used in a wide range of applications thanks to their great textural, mechanical, thermal and electrical properties. Moreover, the versatility of carbon materials facilitates the possibility of modifying on demand their textural, chemical and electrical properties by tuning the carbon precursor as well as by doping the carbon already prepared in accordance with the required applications [1,2,3,4]. Furthermore, due to their chemical stability, the processes can be carried out even if operating conditions are too severe: for example, gas adsorption at a high temperature and pressure or electrochemical applications in an acid or basic medium [5,6,7,8,9,10].

Carbon spheres (CS) are very promising nanostructures in several applications [11,12,13,14]. The spheres can be obtained by different synthesis procedures and methods such as an arc-discharge process, chemical vapour deposition, autoclave methodologies (hydrothermal, solvothermal or by using supercritical fluids) or template methods. Also, several compounds are used as a source of carbon, and among the most commonly used are: resorcinol-formaldehyde (and its derivatives), saccharides or organic acids [15]. Furthermore, the spherical shape allows the entire surface area to be effectively exposed beside the high packing density, which is very important for energy applications [16]. Nevertheless, the synthesis process of CS is usually tedious, and a low amount of material is normally obtained, which makes it an expensive process.

During the last few decades, governments and companies have been making a great effort to improve the current environmental situation. In the research field, one of the strategies involves recycling wastes to obtain advanced materials [17,18,19]. On this subject, the world plastic production almost reached 350 million tonnes in 2017. In 2016, only 8.4 million tonnes of plastic waste were collected in order to be recycled inside and outside the EU [20]. Moreover, 17.5% of total plastic production corresponds to Low-Density Polyethylene (LDPE), which is the main compound in plastic bags, trays, containers and agricultural films.

Previous works have investigated the pyrolysis of different polymers into a closed reactor obtaining carbon spherules [21,22]. However, they did not succeed in obtaining highly graphitic materials, nor have they studied the influence of adding metals.

Taking this data into consideration, we have in the present work prepared carbon microspheres from LDPE (solvent free and under autogenic pressure) through a facile one-step method, which implies a high carbon yield and contributes to the development of graphitic clusters, whereby the conductivity of the material is improved. At the same time, the influence of metal doping on the physical and chemical properties of CS has been also reported.

To our knowledge, there has not been any previous reporting of the synthesis of highly graphitic and magnetic CS nanocomposites prepared in similar conditions.

## 2. Materials and Methods

Three different carbon spheres (CS)-metal composites were obtained by pyrolysis of Low Density Polyethylene (LDPE) (supplied by Sigma-Aldrich) catalyzed by the metal salts Fe(C_2_H_3_O_2_)_2_, Co(C_2_H_3_O_2_)_2_ or Ni(C_2_H_3_O_2_)_2_. Additionally, a metal-free material was obtained under the same experimental conditions for comparative purposes. The samples thus obtained were labelled as PE-Fe, PE-Co, PE-Ni and PE respectively. First, 0.5 *g* of LDPE was placed and treated at 700 °C into a closed hand-made Hastelloy^®^ reactor of 25 mL capacity. The heating rate was 10 °C/min with a dwell time of 2 h at the target temperature. The yield percentage of this synthesis was used to calculate the amount of the corresponding metal precursor to obtain CS-metal composites doped with 10 wt.% of metal.

The amount of metal was determined by a thermogravimetric analysis (TGA), taking into account that the ash percentage corresponds to the metal oxide. TGA was carried in the air with a heating rate of 10 °C/min to 900 °C/min using a Mettler-Toledo TGA/DSC1 thermogravimetric analyzer.

The texture and morphology of the samples were analyzed by scanning electron microscopy (SEM), using a FEI microscope model Quanta 400 and High-resolution transmission electron microscopy (HRTEM) was performed using a FEI Titan G2 microscope. 

The crystalline phases of the obtained materials were analysed by Bruker D8 Venture X-ray diffractometer (BRUKER, Rivas-Vaciamadrid, Madrid, Spain).

Raman spectra were recorded using a Micro-Raman JASCO NRS-5100 dispersive spectrophotometer with a 532 nm laser line.

## 3. Results and Discussion

**Figure 1:**
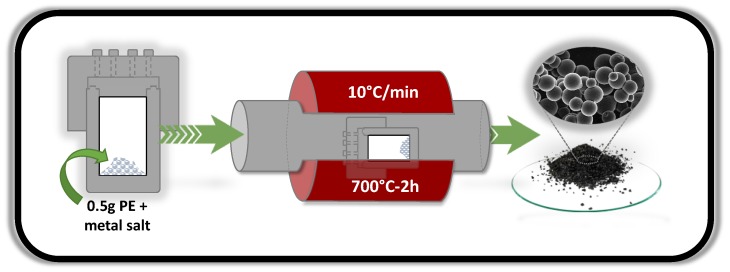
Experimental scheme of the synthesis process.

The schematic diagram outlining the synthesis of the carbon microspheres is illustrated in Figure 1.

The CS-metal composites that were obtained were labelled as PE-X, where “X” stands for the corresponding metallic precursor salt. For a comparison, a metal-free CS material (PE) was prepared under the same experimental conditions. The metal contents for PE-Fe, PE-Co and PE-Ni were 3.0, 7.8 and 6.6 wt.%, respectively.

**Figure 2:**
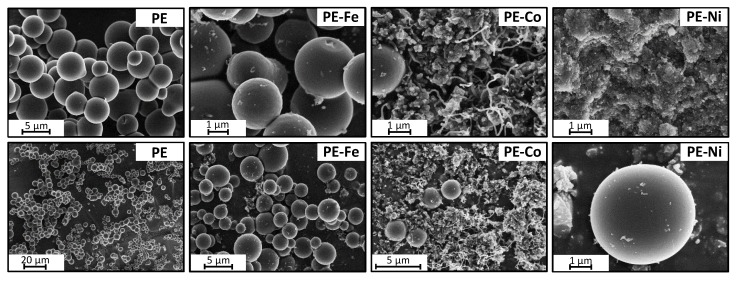
SEM images of carbon spheres nanocomposites at different magnifications.

**Figure 3:**
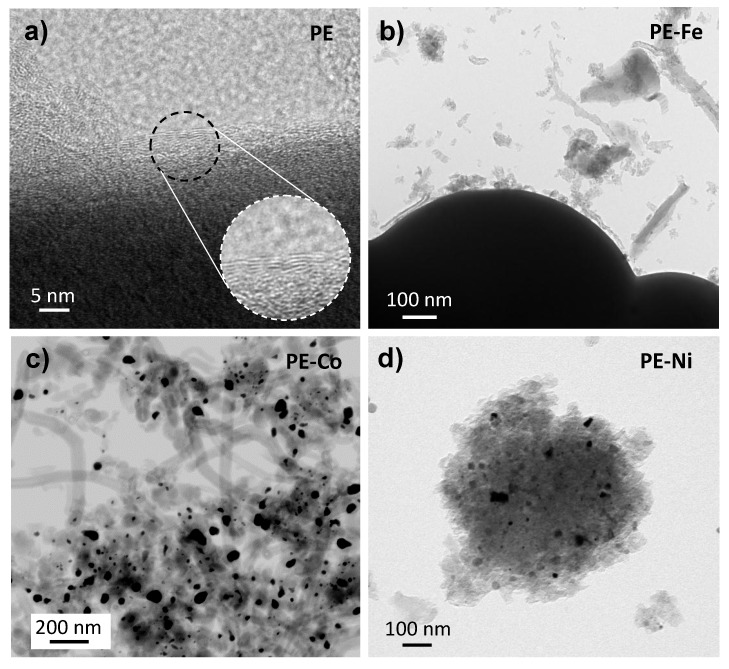
(**a**–**d**) TEM and HRTEM images.

Figure 2 and Figure 3 illustrate the representative SEM and HRTEM images of PE, PE-Fe, PE-Co and PE-Ni. The images reveal the presence of CS in all cases. The pyrolysis of polyethylene under the experimental conditions of temperature (700 °C) and pressure (~70 bar generated in-situ) results in smooth carbon microspheres that are highly homogeneous in size and shape, whereas the presence of metal provokes the development of carbon nanofibers on the spheres-composites which are especially small in the case of the CS prepared with the iron precursor (PE-Fe). The presence of cobalt in the spheres (PE-Co) causes the growth of carbon nanofibers that are significantly longer than the ones obtained using an iron precursor. On the other hand, almost no microspheres remain in the presence of nickel; instead, graphitic nanostructures emerge.

The TEM images (Figure 3) illustrate in detail the graphitization effect on the samples. Figure 3a,b shows quite thick carbon spheres which prevent the penetration of electron beams through them; however, it can be clearly observed that PE also features a graphitic order. The above-mentioned catalytic effect of metal doping is also evidenced by TEM, whilst small carbon nanofibers (CNFs) are surrounding the microspheres in PE-Fe, and the cobalt has further catalyzed the CNFs. On the other hand, Figure 3d reveals Ni nanoparticles embedded on the graphitic cluster. It should also be noted that the metal phase is highly dispersed throughout the carbon matrix with a small particle size.

The spherical shape of these materials could be attributed to the generated pressure during the pyrolysis process. This statement is based on our previous studies related to plastic waste pyrolyzed into an open reactor, where the presence of carbon microspheres was not found [18].

**Figure 4:**
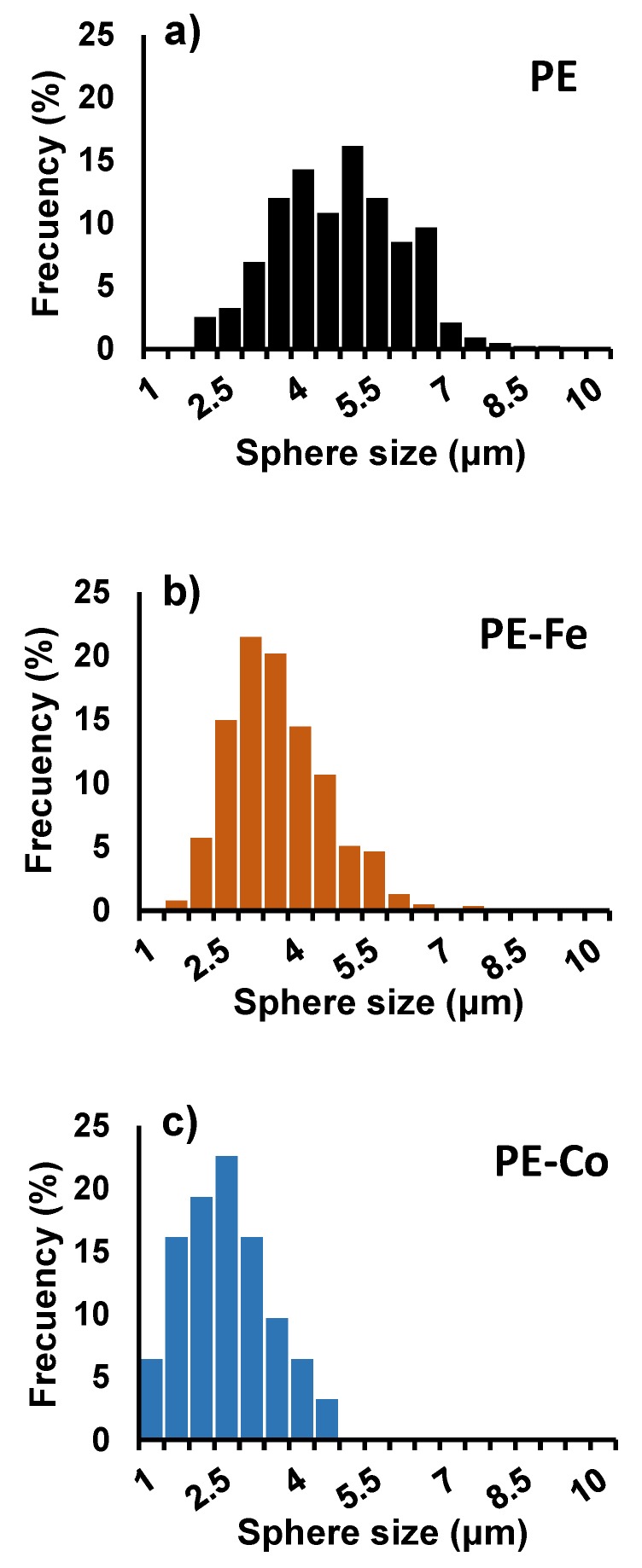
(**a**–**c**) Sphere size distribution.

The sphere size distribution was measured just in those cases where there was a representative statistical population. The obtained histograms are depicted in Figure 4. PE shows a broader distribution than PE-Fe or PE-Co, which is centered on 4.5 μm; however, not only the distribution is narrower for the PE-metal samples; additionally, it is centered on a smaller size. Therefore, the presence of metal catalyzes more homogeneous spheres in terms of size, and furthermore these spheres are smaller; such an effect is slightly accentuated in the case of Co, maybe due to the higher metal content.

**Figure 5:**
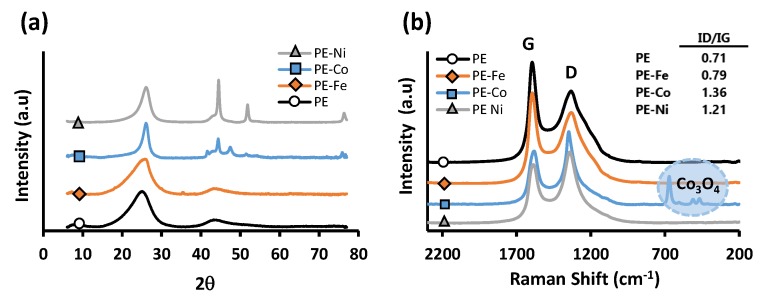
(**a**) XRD patterns (**b**) and Raman spectra.

The presence of graphitic domains in the prepared samples is evidenced by X-ray powder diffraction (XRD) and Raman Spectroscopy (Figure 5a,b respectively). All materials show graphitic structures, especially in the case of PE, in which no metal catalyst of graphitization was added, indicating the effect of the generated pressure once again. The XRD pattern (Figure 5a) shows the typical diffraction peaks of graphitic crystals at 2θ values around 26° and 44.5°, consistent with the JCPDS card 89-8487. The peaks are intense and well defined, indicating a high crystallinity for all of the samples. The carbon matrix maintains Ni and Co in a 0 oxidation state according to the JCPDS cards 04-0850 (Ni fcc), 15-0806 (Co fcc) and 05-0727 (Co hcp). However, no metallic Fe diffraction peak is observed in the XRD patterns, and this fact, coupled with the Raman results (no iron oxide signal was obtained either), leads us to believe that the Fe(0) crystals could have a mean size that is too small or a very slim laminar shape, as a result of which the ultra-small crystal size is below the XRD detection limit [23].

On the other hand, metal-free carbon microspheres (PE) present the highest ordering level, pointed out by an intense and well-defined G band (which is associated with the development of the sp2 carbon structure), and in particular by the small ratio ID/IG, since the D band is an indicator of disorder [24,25]. The intensity ratio ID/IG increases significantly for PE-Co and PE-Ni. These samples are the ones that have grown further graphitic structures as fibers or clusters, so they have also developed a large number of graphenic edges, which belong to the group of defects responsible for the increment of the D band [26]. These data also demonstrate that the samples are endowed with a high electrical conductivity since such a conductivity is improved with the development of sp2 hybridized carbon fractions, which contribute to the G band in Raman spectra. According to the literature, the lower the intensity ratio of the D/G bands, the higher the electronic conductivity [27,28].

**Figure 6:**
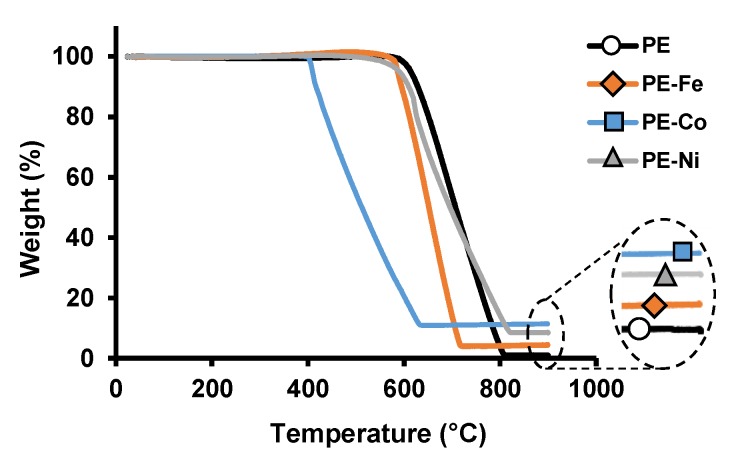
TGA in air. Heating rate of 10 °C min^−1^.

The thermogravimetric analysis (TGA) under air atmosphere (Figure 6) manifests an extraordinary thermal stability for PE-Fe, PE-Ni and especially for PE; all of which remain completely stable until 579, 578 and 615 °C, respectively; those temperatures are comparable to that of graphite [29]. This fact supports the highly ordered graphitic structure mentioned above, since the higher the ordering, the better the thermal stability.

Concerning the PE-Co sample, its thermal stability is significantly lower compared to the others, and this fact could be attributable to the cobalt effect because it catalyses the gasification process further than nickel or iron [30]; besides, the graphitic ordering decreases for PE-Co according to the Raman data. In this regard, as is well known, the stability against oxidation depends on the size, orientation and organization of the graphene layer planes inside the structures [31].

**Figure 7:**
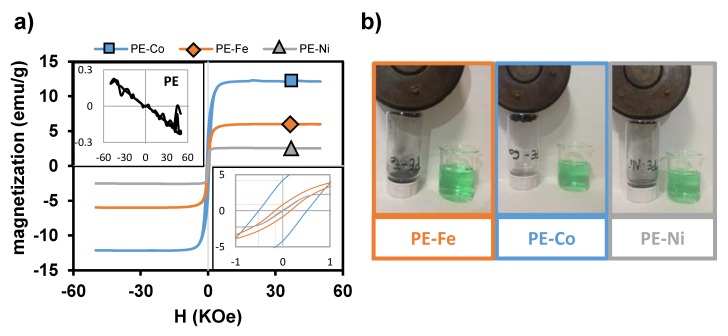
Magnetic behaviour: (**a**) Magnetic susceptibility at 293 K. (**b**) Pictures showing magnetic properties.

The magnetic properties were measured at 293 K using a SQUID QUANTUM magnetometer. The magnetization curve of PE (Figure 7a) exhibits the typical diamagnetic behaviour of carbon polymorphs [32,33], which becomes ferromagnetic by adding metals, as indicated by the hysteresis loop. However, the narrow loop in PE-Ni indicates a superparamagnetic-like behaviour. The saturation of the magnetization follows the order PE-Co > PE-Fe > PE-Ni, reaching values of 12.15, 6.00 and 2.58 (emu/g), respectively. This trend was already noticed by other authors [34,35]. Furthermore, the magnetism is also evidenced by the fact that the CS nanocomposite materials are strongly attracted by a magnet (Figure 7b). This aspect is remarkably interesting, as it enables the separation of the material from the medium upon the application of an external magnetic field.

Consequently, in the view of their properties, these materials would be well suited as electro-catalysts, among other potential applications, but further studies are needed to implement these approaches.

## 4. Conclusions

In summary, we have obtained highly graphitic carbon microspheres that are quite homogeneous in terms of size and shape, through an easy and fast method, where the precursor polymer was pyrolyzed in the presence of autogenic pressure. The doping with transition metals has triggered the growth of carbon nanostructures, with different sizes, around the microspheres, besides conferring them new properties such as magnetism. Therefore, we have succeeded in obtaining CSs with excellent properties (magnetism, a high degree of graphitization, high conductivity or high thermal stability), which make them suitable for a wide range of applications, even under severe conditions.
